# Exploration of Methodologies for Developing Antimicrobial Fused Filament Fabrication Parts

**DOI:** 10.3390/ma16216937

**Published:** 2023-10-29

**Authors:** Sotirios Pemas, Eleftheria Xanthopoulou, Zoi Terzopoulou, Georgios Konstantopoulos, Dimitrios N. Bikiaris, Christine Kottaridi, Dimitrios Tzovaras, Eleftheria Maria Pechlivani

**Affiliations:** 1 Centre for Research and Technology Hellas, Information Technologies Institute, 6th km Charilaou-Thermi Road, 57001 Thessaloniki, Greece; sopemas@iti.gr (S.P.); dimitrios.tzovaras@iti.gr (D.T.); 2 Laboratory of Chemistry and Technology of Polymers and Colors, Department of Chemistry, Aristotle University of Thessaloniki, 54124 Thessaloniki, Greece; elefthxanthopoulou@gmail.com (E.X.); dbic@chem.auth.gr (D.N.B.); 3 Laboratory of General Microbiology, Department of Genetics, Development and Molecular Biology, School of Biology, Aristotle University of Thessaloniki, 54124 Thessaloniki, Greece; konstanto@bio.auth.gr (G.K.); ckottaridi@bio.auth.gr (C.K.)

**Keywords:** additive manufacturing (AM), 3D printing, fused filament fabrication (FFF), filament, antimicrobial properties, *Escherichia coli*, *Staphylococcus aureus*, titanium dioxide (TiO_2_), poly(lactic acid) (PLA), mechanical properties

## Abstract

Composite 3D printing filaments integrating antimicrobial nanoparticles offer inherent microbial resistance, mitigating contamination and infections. Developing antimicrobial 3D-printed plastics is crucial for tailoring medical solutions, such as implants, and cutting costs when compared with metal options. Furthermore, hospital sustainability can be enhanced via on-demand 3D printing of medical tools. A PLA-based filament incorporating 5% TiO_2_ nanoparticles and 2% Joncryl as a chain extender was formulated to offer antimicrobial properties. Comparative analysis encompassed PLA 2% Joncryl filament and a TiO_2_ coating for 3D-printed specimens, evaluating mechanical and thermal properties, as well as wettability and antimicrobial characteristics. The antibacterial capability of the filaments was explored after 3D printing against Gram-positive Staphylococcus aureus (*S. aureus*, ATCC 25923), as well as Gram-negative Escherichia coli (*E. coli*, ATCC 25922), and the filaments with 5 wt.% embedded TiO_2_ were found to reduce the viability of both bacteria. This research aims to provide the optimal approach for antimicrobial and medical 3D printing outcomes.

## 1. Introduction

Fused Filament Fabrication (FFF) technology is one of the most prevalent techniques in additive manufacturing (AM) [1,2,3] that has been developed exponentially [4,5]. This can be attributed to its rapid prototyping capabilities and cost-effective nature [6]. Due to the versatility that it provides, it can be applied for building every type of geometry from a variety of materials. The majority of the materials utilized in additive manufacturing applications consist of polymer-based composite materials and polymer blends [7] with the most commonly used in 3D printing of composite materials being Poly(lactic acid) (PLA) and acrylonitrile butadiene styrene (ABS) [8,9].

Poly(lactic acid) (PLA), a biobased and compostable polyester, finds diverse applications in tissue engineering, drug delivery, and medical applications [10,11,12]. It has been proven that when polymers are combined with specific nanoparticles, such as TiO_2_, they acquire antimicrobial properties [13,14]. Titanium dioxide (TiO_2_) has the ability to tune polymer properties such as antimicrobial activity, UV resistance, opacity, gas barrier, and color stability [7,15].

Numerous efforts have been documented in the literature with the aim of creating antimicrobial filaments for Fused Filament Fabrication (FFF) technology. Vidakis et al. [7] published a study that used (TiO_2_) nanoparticles as nanofillers in order to enhance the properties of polypropylene (PP). The findings demonstrated that the characteristics (mechanical properties) of the nanocomposites had improved and it was demonstrated that PP/TiO_2_ could be a nanocomposite system for use in AM applications. González et al. [16] examined PLA filled with TiO_2_ nanoparticles in various particle content concentrations in a bacterial culture of *E. coli* and found that TiO_2_ nanoparticles decreased the amount of extracellular polymeric substance and reduced bacterial growth. Also, no significant differences were observed for higher contents than 1% TiO_2_ nanoparticles.

However, while most studies suggest that 3D-printed parts can be produced using antimicrobial filaments containing additives like TiO_2_ with antimicrobial properties, this study examines a coating methodology to determine if it offers comparable results to the development of antimicrobial filaments. Implementing this coating technique in 3D-printed PLA components offers an alternative approach to imparting antimicrobial properties, bypassing the need to develop antimicrobial composite filaments for subsequent part fabrication through 3D printing. Although coating methodology has been used in scaffold applications to improve cell attachment/proliferation [17], here it is proposed as a method to provide antibacterial properties to medical 3D-printed parts and components or daily devices used in agri-food sector [6,18].

This study introduces an innovative approach to producing antimicrobial 3D-printed components through FFF AM technology. For the experiments, PLA was selected as the matrix material due to its biobased nature, as opposed to petroleum-based alternatives [19], and its favorable mechanical properties [20]. Given that FFF technology employs feedstock materials in filament form, the integration of TiO_2_ nanoparticles into the filament is pursued to achieve antibacterial properties. The inclusion of Joncryl in small quantities as a chain extender enhances PLA’s printability and mechanical properties by elevating molecular weight, complex viscosity, and melt flow index [21,22,23].

This work compares two methodologies for developing antibacterial 3D-printed components. The first method involves developing a composite filament that combines PLA, TiO_2_, and Joncryl, suitable for use in all FFF 3D printers. A reference point is established by developing an additional filament made of PLA and Joncryl alone. The second method entails a coating process (dispersion immersion method) applied to the final parts manufactured using the PLA and Joncryl filament. These filaments were developed to produce specimens as a proof of concept for the future production of DIY (do-it-yourself) customized or pre-existing antimicrobial parts, medical tools, and more. All three specimen categories (1. PLA/Joncryl filament, 2. PLA/Joncryl/TiO_2_ filament, and 3. PLA/Joncryl filament with a coating process) underwent comprehensive analysis, including physiochemical characterization and mechanical and antibacterial property assessments.

**Figure 1:**
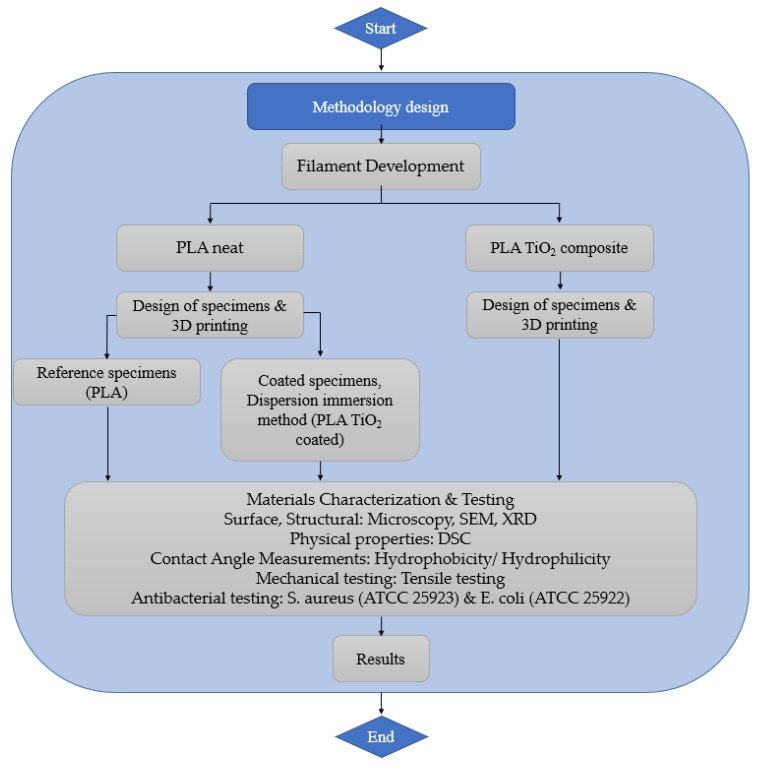
Architectural diagram.

This paper is organized as follows: Section 2 outlines the materials and methods employed in the development of the present study. Section 3 encompasses the results, focusing on the characterization of the developed filaments and the coating methodology and presents data concerning their antibacterial activity and mechanical properties. Finally, the study concludes in Section 4. Figure 1 presents the architectural diagram of the methodology steps that were followed. These steps include the development of the filaments and the necessary tests conducted to extract results for characterizing the properties of the 3D-printed specimens.

## 2. Materials and Methods

### 2.1. Materials

In this study, the experimental materials used for the development of the filament included PLA in pellet form, Joncryl as a chain extender, and titanium dioxide (TiO_2_) nanoparticles. The PLA pellets used were of PLA 4043D type, supplied by 3devo (Utrecht, The Netherlands). The chain extender Joncryl ADR^®^ 4400 was supplied by BASF (Ludwigshafen, Germany). It possesses an epoxy equivalent weight of 485 g/mol and a weight-average molecular weight of 7100 g/mol. Aeroxide^®^ TiO_2_ P25 with a nanoparticle size of 21 nm and a specific surface area of 35–65 m^2^/g was supplied from Sigma-Aldrich (Saint Louis, MO, USA).

### 2.2. Development of a PLA-Based TiO_2_ Filament

In order to develop the two distinct filaments based on PLA pellets, the PLA pellets were vacuum-dried overnight at 40 °C. The dried PLA was subsequently mixed with Joncryl to formulate the PLA/Joncryl filament (named as PLA), and with both Joncryl and TiO_2_ to fabricate the potentially antimicrobial PLA/Joncryl/TiO_2_ filament (named as PLA TiO_2_ comp). Table 1 provides the composition of each filament. In total, 2 wt.% of Joncryl was used, and it was proven by Grigora et al. [24] in a previous work that it gives the best physicochemical properties to the final filament.

The filaments were fabricated using the 3devo Composer Series 350/450 filament maker (Utrecht, The Netherlands). Parameters were configured for extrusion of a 1.75 mm diameter filament. However, due to variations, the filament thickness deviation was ±0.06, resulting in a final filament diameter ranging between 1.69 mm and 1.81 mm. The machine contains a mixing screw that aids in material passage through four heating zones. Upon melting, the material is extruded as filament through a nozzle.

**Figure 2:**
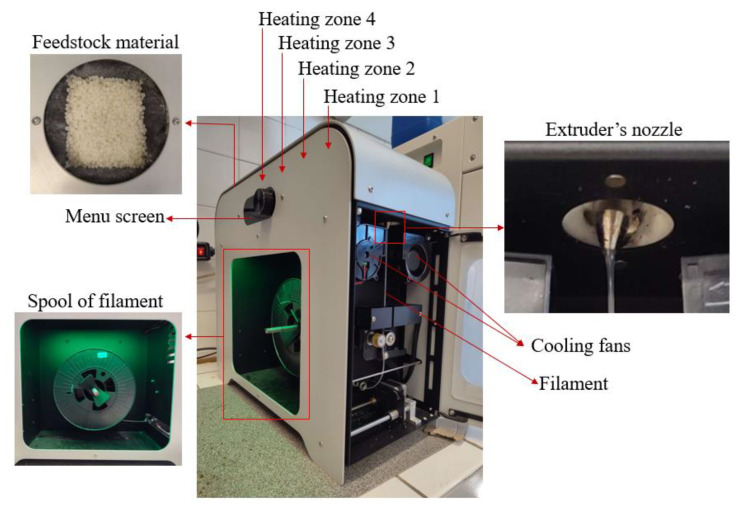
3devo Filament Maker Overview.

The four heating zones of the extruder can be independently set to distinct temperature values. In this experiment, temperature ranged from 175 °C to 192 °C. Various combinations were tested to optimize filament extrusion. Ultimately, the optimal temperature combination was found to be Heater 1—180 °C, Heater 2—192 °C, Heater 3—187 °C, and Heater 4—175 °C. Heater 1 is closest to the nozzle, while Heater 4 is situated near the hopper. The extruder’s screw rotational speed, driving material through the heating zones and extruder, was set at 4.1 rpm. For filament cooling, integrated fans were adjusted to 60%, facilitating timely solidification for spool collection during fabrication. Figure 2 illustrates the characteristic components of the 3devo filament maker.

### 2.3. Fabrication of 3D-Printed Specimens

After the successful development of both filaments, the FFF technology was used to fabricate the specimens. All specimens were designed using SOLIDWORKS^®^ CAD Software (2022 SP2.0 Professional version) and manufactured utilizing an Original Prusa i3 MK3S+ 3D printer. Each part’s 3D printing parameters were established using Prusa Slicer 2.5.0 software. For the PLA/Joncryl filament, the nozzle temperature was set at 220 °C, while for the PLA/Joncryl/TiO_2_ filament, it was set at 240 °C. The bed temperature for both filaments was set at 60 °C. In all cases, a 0.4 mm nozzle and 0.2 mm layer height were employed. The specimens from both filaments were printed with a 100% fill density and concentric fill pattern for infill. Settings not explicitly mentioned in the FFF process were maintained at their default values as per the Prusa Slicer software^TM^ (Version 2.6.1) utilized in this study. Table 2 presents the specimens, along with their dimensions, used in the present study to characterize the properties of the 3D-printed specimens fabricated with the developed filaments. 

**Figure 3:**
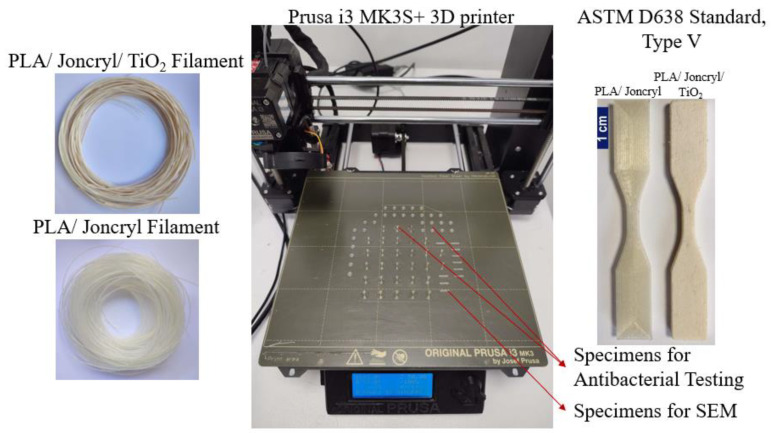
3D Printer and Printed Specimens Visual Overview.

The dimensions of the specimens were determined by the requirements of the testing equipment: Φ 5 mm × 1 mm for antibacterial testing and 10 × 2 × 1 mm for Scanning Electron Microscopy (SEM). Figure 3 displays the filament reels for the Prusa 3D printer, used to produce these specific specimens in two different surface configurations. Notably, the Φ 5 mm × 1 mm specimens provided satisfactory results, making the larger surface specimens redundant for antibacterial testing.

### 2.4. Coating Methodology: Dispersion Immersion Method

**Figure 4:**
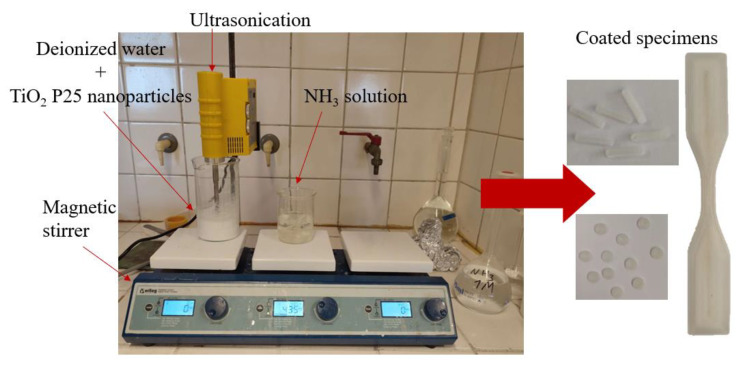
Coated specimens: materials and procedures used for dispersion immersion method.

Between the two developed filaments, the PLA/Joncryl filament was selected for 3D printing the specimens that subsequently underwent a dispersion immersion coating process (as shown in Figure 4) to confer antimicrobial properties. 

For the coating procedure, initially, the samples were immersed in a 1 M aqueous ammonia (NH_3_) solution (pH = 11.5) and subjected to magnetic stirring at a speed of 435 rpm for 4 h at room temperature. Simultaneously, a 2 wt.% aqueous dispersion of TiO_2_ was prepared. This dispersion underwent magnetic stirring for 1 h at a speed of 950 rpm, to prevent sedimentation in the stirring vessel. Around 30 min prior to removing the samples from the ammonia solution, an ultrasonication process was initiated using an ultrasonication probe. The process involved alternating cycles of 2 min of ultrasound treatment followed by 2 min of rest, repeated for a total of 6 cycles.

After removal from the ammonia solution, the samples were thoroughly rinsed with deionized water and subsequently dried in an oven at 55 °C for 30 min. The dispersion containing deionized water and TiO_2_ was heated at 70 °C and stirred magnetically for 30 min, with a stirring speed of 1200 rpm. Following this, the samples were immersed in this dispersion. The TiO_2_ dispersion (with the specimens immersed) was heated to 70 °C and stirred magnetically at a speed of 875 rpm for a duration of 2 h. Afterwards, it was sonicated for 10 min, while maintaining the temperature at 70 °C. Finally, the samples were washed with ethanol and placed in an oven set at 75 °C for 10 min to facilitate drying. Upon completion of the aforementioned steps, the coating process concluded, resulting in the adhesion of TiO_2_ powder particles to the samples (named as PLA TiO_2_ coated).

### 2.5. Materials Characterization

#### 2.5.1. Microscopy

The filaments’ morphological features were examined with a stereoscope. Images were taken using a Jenoptik (Jena, Germany) ProgRes GRYPHAX Altair camera attached to a ZEISS (Oberkochen, Germany) SteREO Discovery V20 microscope and Gryphax image capturing software was used.

#### 2.5.2. Scanning Electron Microscopy (SEM)

Scanning Electron Microscopy (SEM) images were captured using a JEOL (Tokyo, Japan) 2011 (JMS-840) electron microscope, equipped with an Oxford (Abingdon, UK) ISIS 300 energy-dispersive X-ray (EDX) micro-analytical system. Every specimen was positioned on the holder and coated with carbon to enhance the conductivity for the electron beam. The images were taken under an accelerating voltage of 2 kV, a probe current of 45 nA, and a counting time of 60 s.

#### 2.5.3. X-ray Diffraction (XRD)

X-ray Diffraction (XRD) analyses of the polymers and copolymers were executed across a 2θ range of 5 to 80°, at intervals of 0.05°, and a scanning speed of 1.5 deg/min. The assessments were conducted using a MiniFlex II XRD system from Rigaku Co. (Tokyo, Japan) with Cu Ka radiation (λ = 0.154 nm).

#### 2.5.4. Differential Scanning Calorimetry (DSC)

A PerkinElmer Pyris DSC-6 differential scanning calorimeter, which was calibrated with pure indium and zinc standards, was employed for the analysis. Samples of 5 ± 0.1 mg sealed in aluminum pans were used and all experiments were performed under N_2_ atmosphere with a flow rate of 20 mL/min. Each specimen was subjected to a heating process from room temperature to 200 °C at a pace of 20 °C/min, then cooled down to 25 °C at the same rate of 20 °C/min and reheated to 200 °C at a 20 °C/min. The degree of crystallinity (X_c_) was determined using Equation (1):

(1) $$\mathrm{Xc}\left(\%\right)=\left(\frac{{\Delta H}_{m}-{\Delta H}_{\mathrm{cc}}}{{\Delta H}_{f}^{0}-\frac{1-\mathrm{wt}.\%\;\mathrm{additive}}{100}}\right)\times 100$$

where ΔH_m_, ΔH_cc_, and ΔH_f_^0^ are the experimental melting enthalpy, the cold crystallization enthalpy, and the theoretical heat of fusion of 100% crystalline PLA (ΔH_f_^0^ = 93 J/g), respectively.

#### 2.5.5. Contact Angle Measurements

The water contact angle (WCA) was assessed with the Ossila (Sheffield, UK) Contact Angle Goniometer L2004A. The analysis of WCA for the samples was conducted through the sessile drop technique. A quantity of 25 μL of distilled water was delicately placed atop the surface of the 3D-printed plates (*n* = 3) and scaffolds. High-resolution images were captured within a span of 20 s and further analyzed using the Ossila Contact Angle Software v3.1.1.0. The statistical evaluation was conducted through a one-way ANOVA followed by a post hoc Tukey test, facilitated by the GraphPad Prism 6 software. A *p*-value of less than 0.05 was deemed as indicative of statistical significance.

#### 2.5.6. Tensile Testing

Tensile testing evaluations were conducted utilizing a Shimadzu EZ Test Tensile Tester, Model EZ-LX, equipped a with a 2 kN load cell, following the ASTM D638 standards at a crosshead speed of 5 mm/min. For the testing, 3D-printed dumb-bell-shaped tensile type V test specimens were employed. Each sample underwent at least five separate assessments with the resulting data averaged to derive the mean values for Young’s modulus, stress at break, and elongation at break. Data analysis was performed using one-way ANOVA, followed by a post hoc Tukey test, facilitated by GraphPad Prism 6 software. A *p*-value under 0.05 was established as the threshold for statistical significance.

#### 2.5.7. Antibacterial Testing Methodology

Gram-positive Staphylococcus aureus (*S. aureus*, ATCC 25923), as well as Gram-negative Escherichia coli (*E. coli*, ATCC 25922) single colonies were inoculated in 10 mL of freshly prepared Nutrient Broth and incubated with agitation until reaching an OD600 measurement equal to 0.3–0.5. Then, 2 mL of the culture was moved to a microcentrifuge tube, spun for 1 min at 10.000 g, and the supernatant was removed. The cell pellet was resuspended in 2 mL PBS and spun for 1 min at 10.000 g. PBS washing was repeated twice. After the last spin, the pellet was resuspended in 2 mL PBS and 500 µL was transferred in four different glass flasks containing 4.5 mL PBS. Serial dilutions of each flask were transferred on Nutrient Agar plates using a bent glass pipette and incubated overnight at 37 °C to determine the initial cfu/mL for each flask. After transferring to the plates, a control filament was added in one of the flasks, a TiO_2_-based filament was added to the second, a filament coated with TiO_2_ was added to the third, whereas the last flask was used as a no filament control. The flasks were incubated for 1 h at 37 °C and 250 rpm, and then serial dilutions for each flask were transferred to fresh Nutrient Agar plates again. This procedure was repeated after one more hour of the flasks’ incubation. The plates were left to incubate overnight at 37 °C. The number of colonies that represent the surviving bacteria was counted the following day and the possibility of antibacterial activity of the filament was determined.

The filaments’ antibacterial effectiveness against *S. aureus* and *E. coli* is reported as the mean standard deviation (SD) after 60 and 120 min of contact. Each experimental procedure was replicated three times (n = 3) for each bacterium strain. For statistical analysis, two-way ANOVA with repeated measurements was performed.

## 3. Results and Discussion

### 3.1. Scanning Electron Microscopy (SEM) and Optical Microscopy

**Figure 5:**
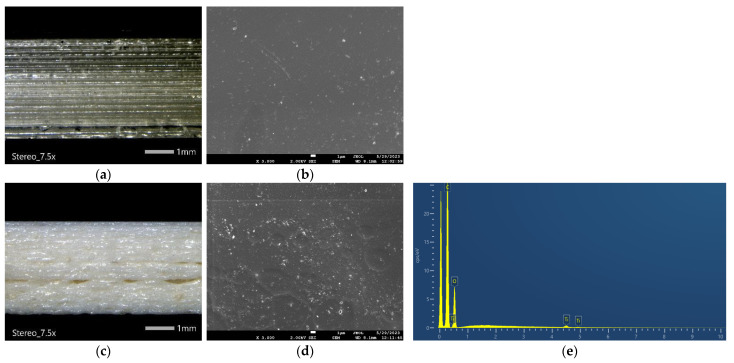

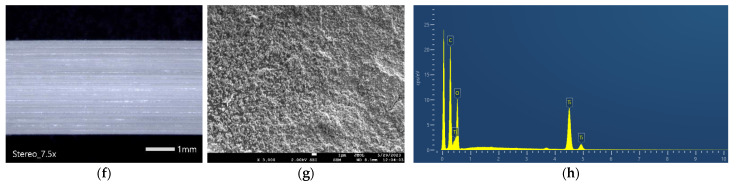
(**a**,**b**) Optical microscopy and SEM image of 3D-printed PLA (PLA/Joncryl filament), (**c**,**d**) optical microscopy and SEM image of 3D-printed PLA TiO_2_ comp (PLA/Joncryl/TiO_2_ filament), (**e**) EDX spectrum of 3D-printed PLA TiO_2_ comp, (**f**,**g**) optical microscopy and SEM image of 3D-printed PLA TiO_2_ coated, and (**h**) EDX spectrum of 3D-printed PLA TiO_2_ coated.

The morphological characteristics and the dispersion of the TiO_2_ nanocomposites in the polymer matrices are examined via microscopic techniques. Figure 5 displays the side surface of randomly selected 3D-printed tensile test specimens, providing a quantitative assessment of interlayer fusion, interlayer defects, or possible inhomogeneities.

It can be observed in Figure 5a,c,f that the 3D printing process utilizing the PLA/Joncryl/TiO_2_ filament does not yield the same 3D printing quality as the PLA/Joncryl filament. This difference arises from the formation of TiO_2_ nanoparticles’ agglomerates, resulting in inconsistent material extrusion flow. This phenomenon is strongly associated with the dimension and the weight fraction of the inorganic additive in the polymer matrix. Specifically, it was found that >1 wt.%. of additive led to an increased agglomeration, and thus, intense surface roughness [26,27,28]. Furthermore, the processing method seems to affect the morphological features of the final specimen. Specifically, the dispersion of TiO_2_ particles is observed to be more homogeneous in the case of the composite, resulting in a smooth surface. On the contrary, the coating procedure led to a surface with augmented roughness, due to the increased percentage of the TiO_2_, as it was verified from the EDX analysis (Figure 5e,h).

### 3.2. X-ray Diffraction (XRD) Measurements

**Figure 6:**
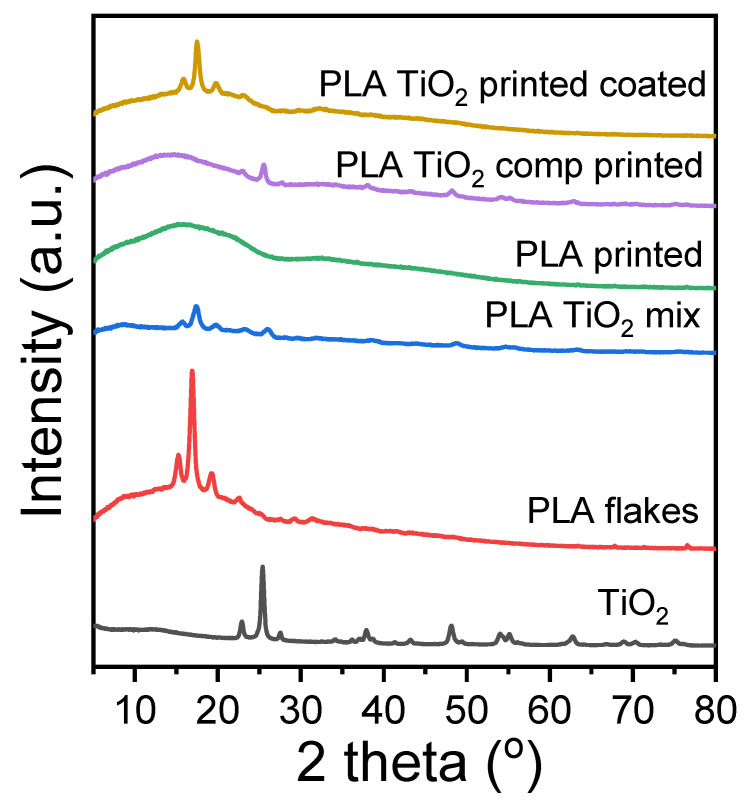
X-ray diffraction patterns of PLA, TiO_2,_ and the 3D-printed specimens.

XRD patterns of the fabricated materials are presented in Figure 6. The characteristic diffraction peaks of PLA appeared at 2θ = 14.8°, 16.5°, 19°, and 22° resulting from the crystal planes (010), (200/110), (203), and (210) [29]. No diffraction peaks appeared in any sample, as thermal processing, such as extrusion and printing, usually leads to amorphous materials [30]. The small crystalline peaks that the coated material exhibits are probably due to the heating during the coating procedure, facilitating some cold crystallization.

### 3.3. Differential Scanning Calorimetry (DSC) Measurements

**Figure 7:**
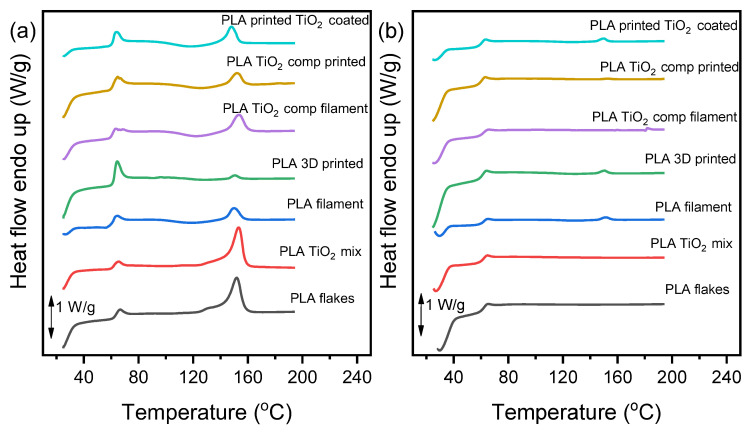
DSC graphs of the materials during heating with rate 20 °C/min, (**a**) first heating, and (**b**) second heating scan.

The thermal properties of the fabricated PLA/TiO_2_ materials were determined using DSC analysis. The recorded DSC thermograms upon the first and the second heating scan are presented in Figure 7. The characteristic thermal transitions, including glass transition temperature (T_g_), cold crystallization temperature (T_cc_), and melting point (T_m_), as well as the % degree of crystallinity of the materials, are summarized in Table 3. No crystallization peak was observed in any of the samples upon cooling from the melt. As can be observed, all the printed samples exhibited a very low degree of crystallinity, as processing methods, such as printing, tend to erase the matrix crystallinity [30]. All samples were amorphous, showing glass transition at 60–62 °C, cold crystallization, and subsequent melting. This observation is in agreement with the obtained XRD patterns. Furthermore, a small reduction in the T_g_ values of the printed samples in comparison to the PLA flakes can be attributed to some degradation during the thermal processing of 3D printing [31]. The coating process seems to also have an effect on the thermal transitions of the final sample, as T_g_ and T_m_ values decreased. However, the thermal transitions of the samples were not significantly affected.

### 3.4. Contact Angle Measurements

**Figure 8:**
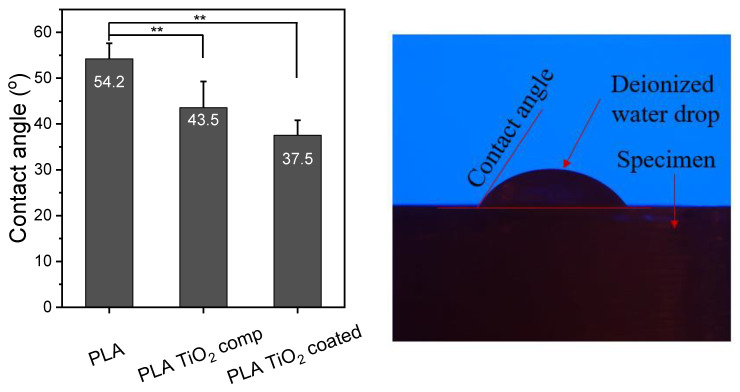
Water contact angle of 3D-printed PLA, PLA TiO_2_ comp, and PLA TiO_2_ coated. One-way ANOVA, ** *p* 0.001–0.01.

The contact angle of the surface with water plays a key role in the characterization of a material, as it can offer an insight into its absorption and its adhesion profile [32]. The water contact angle of polymeric materials is a function of their chemical composition and surface properties (roughness, heterogeneity, and preparation method), as well as temperature [33]. The hydrophilicity of the PLA/Joncryl (PLA), PLA/Joncryl/TiO_2_ (PLA TiO_2_ comp), and PLA/Joncryl coated (PLA TiO_2_ coated) printed specimens was assessed through water contact angle measurements, and the methodology is illustrated in Figure 8. As can be observed, PLA/Joncryl specimens had a contact angle of ~54.2°. Neat PLA appeared less hydrophobic than expected because of its surface roughness. The addition of TiO_2_ to PLA caused a statistically significant decrease in the contact angle values of both the composite and the coated specimen, and a decreasing trend in the values of the composite to the coated sample. This trend is a result of the increased free energy and the increased roughness of the specimen, as can be assumed from the corresponding SEM images [34]. Furthermore, the hydrophilicity of the incorporated TiO_2_ nanoparticles, owing to the unsaturated reactive hydroxyl (-OH) groups, resulted in the significantly enhanced hydrophilicity of the final samples [35,36]. Although the hydrophilic character of a sample is related to its poor resistance to water, this may be beneficial for the contact between the microbial cells and the film which can facilitate the antimicrobial activity.

### 3.5. Tensile Testing Measurements

**Figure 9:**
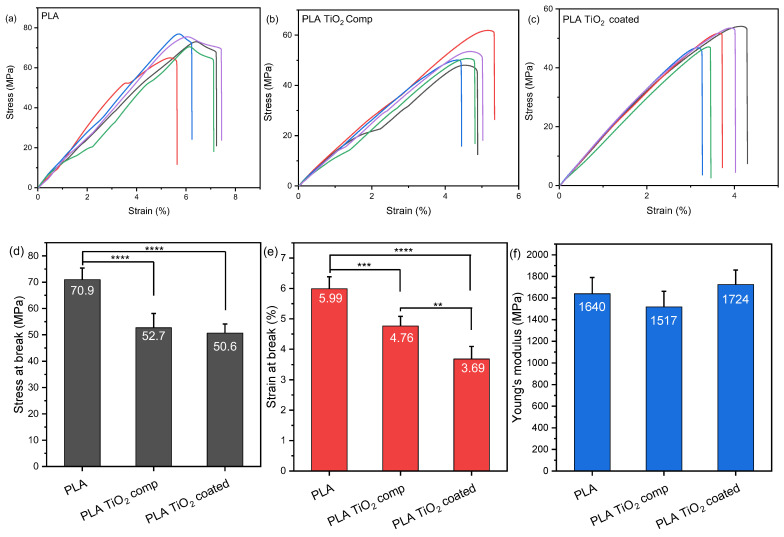
Stress–strain curves of (**a**) PLA and (**b**) PLA TiO_2_ comp printed specimens, (**c**) stress at break, (**d**) strain at break, and (**e**) Young’s modulus values. One-way ANOVA. ** 0.001 < *p* < 0.01, *** 0.0001 < *p* < 0.001, **** *p* < 0.0001.

As the mechanical features of a polymer define its final applications, tensile tests were performed and the results are presented in Figure 9. In Figure 9a–c, stress–strain diagrams of the three specimen categories demonstrate differences in mechanical properties between specimens printed with PLA/Joncryl (PLA) filament, PLA/Joncryl/TiO_2_ (PLA TiO_2_ comp) filament, and specimens printed with PLA/Joncryl filament followed by the coating process (PLA TiO_2_ coated). The PLA specimens exhibit the highest stress and strain at break, whereas the PLA TiO_2_ coated specimens show the highest Young’s modulus value. The stress–strain curves indicate that both the integration of TiO_2_ nanoparticles and the coating process, aimed at providing antimicrobial properties to the final polymeric material, influence the mechanical properties of the developed filament. This is evident in the stress–strain curves, particularly in the ultimate tensile strength (UTS) or the maximum stress the material can endure before failure. The stress–strain curves of the PLA/Joncryl filament demonstrate the highest ultimate tensile strength. As Figure 9d–f illustrates, there is a statistically significant decrease in tensile strength and strain values among the PLA and either composite. The decreased values of the composite sample can be a result of defects on the printed structure (as shown in Figure 5c). The lower values of tensile strength and strain that the coated sample exhibits, in combination with its increased Young’s modulus value, can be attributed to it being soaked in aqueous solution and ammonia, which could have caused some hydrolytic degradation to the material. However, the variation of the Young’s modulus values between all samples was insignificant (*p* > 0.05).

Generally, the percentage and diameter of the particles incorporated in the matrix strongly affect its final features. It has been reported that the weight fractions of TiO_2_ nanoparticles up to 1% increased the tensile strength and strain values, while at high TiO_2_ concentrations, the self-networking of nanoparticles can take place [31]. This phenomenon may lead to the agglomeration and non-homogeneous dispersion of the particles [34]. This could potentially indicate that the agglomeration of TiO_2_ nanoparticles at high loadings may contribute to the weakening of the mechanical properties, as evidenced in the stress–strain diagram, but it is necessary to achieve antimicrobial properties. Agglomerated nanoparticles restrict the interfacial area between themselves and the polymer matrix, resulting in a non-uniform particle distribution and a reduction in the nanoparticle concentration within the composite. Furthermore, it is found that the functionalization of TiO_2_ or the addition of a plasticizer is essential for the improvement of the mechanical features of PLA/TiO_2_ materials compared to the neat PLA [37,38,39]. Similar behavior has been observed with the presence of other metal-based nanoparticles, where concentrations above 1% wt. showed decreased mechanical properties [40,41,42,43].

All things considered, the diagrams in Figure 9 reveal that the stress at break (MPa) exhibits a decrease of 25.67% in the specimens printed with the PLA/Joncryl/TiO_2_ filament compared to those printed with the PLA/Joncryl filament. Moreover, an additional decrease of 3.98% is observed in specimens printed with the PLA/Joncryl filament and subsequently subjected to a dispersion immersion coating process. A similar reduction is noticed in the strain at break (%) with a decrease of 20.53% when comparing the PLA/Joncryl to PLA/Joncryl/TiO_2_ filament and an additional 22.47% decrease after the coating process.

### 3.6. Antibacterial Testing Measurements

**Figure 10:**
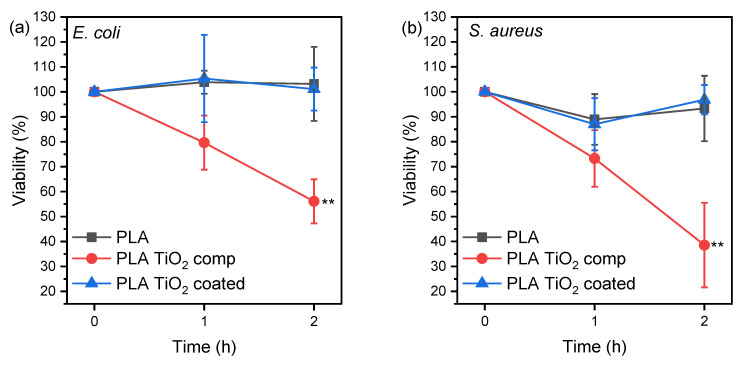
% Viability of bacteria (**a**) *E. coli* and (**b**) *S. aureus* after incubation with PLA, PLA TiO_2_ comp, and PLA TiO_2_ coated specimens. Two-way ANOVA with repeated measurements, ** 0.001 < *p* < 0.01.

In Figure 10, the *x*-axis represents the three time points when the cfu/mL was counted (i.e., 0 h, 1 h, and 2 h), while the *y*-axis represents the % average viability for each bacterium incubated with the different specimens.

The average % viability is calculated as follows: First, the percentage of viability for each replicate is determined by dividing the cfu/mL of each hour by the cfu/mL at hour 0 and then multiplying by 100. Consequently, for each hour, three percentages of viability were obtained (one for each replicate), which were used to calculate the average % viability. These values were used to create the graph.

The results in Figure 10 indicate that the incubation of both bacteria species with the PLA TiO_2_ comp specimen caused a statistically significant reduction over time in the microbes’ viability compared to PLA and PLA TiO_2_ coated specimens. There was no effect on the bacteria strain observed. TiO_2_ is hydrophilic, and thus it decreased the water contact angle of PLA TiO_2_ comp, but it is also antimicrobial, indicated by its generation of reactive oxygen species when it is exposed to light, which oxidize the cytoplasm of bacteria.

TiO_2_ coatings imparted antimicrobial activity when dip-coated on PMMA [44], and their lack of efficiency herein could be due to the leaching of TiO_2_ particles from the PLA TiO_2_ coated specimen, resulting in a low ion concentration in the incubation medium. Concentration is the most important parameter that affects bacteria survival rate on plastic/metal oxide nanoparticles and the 5 wt.% of TiO_2_ added in the PLA TiO_2_ filament was effective [45]. Thus, directly adding metal oxides such as TiO_2_ is an easy and efficient way of additive manufacturing antimicrobial objects.

## 4. Conclusions

In this study, we developed two different filaments for FFF feedstock material. One filament is developed for immediate use in 3D printing antibacterial parts, while the other is intended for 3D printing parts and subsequently providing them with antibacterial properties through a coating process. To ensure perfect repeatability in our experiments and the fabrication of all specimens under exactly the same parameters, the stability of all 3D printing parameters was maintained. Deviating from these parameters would lead to different results. To assess the mechanical performance and antibacterial activity of parts manufactured using these two methods, we conducted a series of tests to draw conclusions regarding their structure and properties. Although SEM and optical microscopy images confirmed the presence of TiO_2_ nanoparticles, antibacterial tests demonstrated that only the parts manufactured by PLA/Joncryl/TiO_2_ filament exhibited significant antibacterial properties. Interestingly, the coated parts exhibited non-antibacterial activity similar to those printed with the control PLA/Joncryl filament, while the PLA/Joncryl/TiO_2_ filament specimens displayed notable antibacterial activity. Consequently, the coating methodology is not recommended, as it does not yield the desired results and involves a more complex procedure requiring access to chemical laboratories, substances, and equipment. In terms of the mechanical attributes of the printed components, the findings indicate that introducing TiO_2_ nanoparticles undermines the mechanical properties, leading to the formation of agglomerates. Future studies should investigate the optimal pretreatment method for the materials and the ideal quantity (wt.%) and size of nanoparticles to achieve the best particle distribution and enhance mechanical properties while maintaining antibacterial characteristics.

Given access to the necessary equipment for filament development, this study proposes a DIY method for creating customized antibacterial parts suitable for production on any commercial FFF 3D printer. These parts could include plastic surfaces for various applications in hospitals, medical tools, laboratory plastic parts like Petri dishes, plastic centrifuge test tubes, and pipette tips for cases where reusable antibacterial plastics are applicable. They can also be used in research DIY devices such as bioreactors, orbital shakers, or other costly components that can be replaced with this cost-effective 3D printing solution. Finally, with further research and possible improvements, this proposed filament could be assessed for 3D printing implants or scaffolds to be used in bone reconstruction applications, potentially replacing existing materials used in these applications.

## Figures and Tables

**Table 1:** Summary of Fabricated Filaments.

Composite Filaments	Experimental Materials		
	PLA (wt.%)	TiO_2_ (wt.%)	Joncryl (wt.%)
PLA/Joncryl	98%	-	2%
PLA/Joncryl/TiO_2_	93%	5%	2%

**Table 2:** 3D-Printed Specimens.

Type of Test	Dimensions of 3D-Printed Specimens
Scanning Electron Microscopy (SEM)	10 × 2 × 1 mm
Tensile	ASTM D638 Standard, Type V [25]
Antibacterial	Φ 5 mm × 1 mm

**Table 3:** Thermal characteristics of the samples as measured by DSC.

Sample	1st Heating				2nd Heating			
	T_g_	T_cc_	T_m_	X_c_	T_g_	T_cc_	T_m_	X_c_
PLA flakes	61.9	-	151.9	33.5	60.7	-	-	0
PLA TiO_2_ mix	60.7	-	153.4	35.7	60.5	-	-	0
PLA filament	60.3	118.5	149.8	1.4	63.3	129.1	151.1	0
PLA 3D printed	61	127	150.7	0.0	59.7	127.7	150.7	0
PLA TiO_2_ comp filament	59.9	121.7	153.3	3.0	60.5	-	-	0
PLA TiO_2_ comp printed	61.8	124.7	152	1.1	58.9	-	152.7	0.1
PLA TiO_2_ coated printed	60.1	116.8	148.1	0.5	58.9	125.8	143.1	0

## Data Availability

All the data are included in the manuscript.
